# Complete Genome Sequence of Enterobacter xiangfangensis Pb204, a South African Strain Capable of Synthesizing Gold Nanoparticles

**DOI:** 10.1128/MRA.01406-18

**Published:** 2018-12-06

**Authors:** Nicholas Ryan Ho, Kulsum Kondiah, Pieter De Maayer

**Affiliations:** a School of Molecular and Cell Biology, University of the Witwatersrand, Johannesburg, South Africa; b Department of Biotechnology and Food Technology, University of Johannesburg, Johannesburg, South Africa; Loyola University Chicago

## Abstract

Enterobacter xiangfangensis Pb204, isolated from acid mine decant from a uranium mine, produces a wide variety of gold nanoparticles (AuNPs), ranging from large triangular plates to small spherical AuNPs. The complete genome sequence of this isolate incorporates an integrative and conjugative element which may be pivotal to AuNP synthesis.

## ANNOUNCEMENT

Mining operations, which often have both high concentrations of heavy metal ions and low pH conditions, harbor a range of specialized bacteria which can survive under these conditions (1). A strong correlation has been found between metal resistance mechanisms in such bacteria and their ability to synthesize metallic nanoparticles (NPs) (2). These bacterially synthesized NPs have the potential to be used in several biotechnological applications, including the detection of narcotics, drug delivery, and the treatment of cancer (3, 4). Bacterially synthesized NPs are of particular interest given their low production cost and low impact on the environment (5). A gold nanoparticle (AuNP)-producing bacterium, Enterobacter xiangfangensis Pb204, was cultured from acid mine decant released from a uranium mine in South Africa (26°06′26.8″S, 27°43′20.2″E).

The strain was cultured on Luria-Bertani (LB) agar and incubated at 37°C for 24 h. Genomic DNA was extracted with the Promega Wizard genomic DNA purification kit per the manufacturer’s instructions. The sequencing library was prepared with the Nextera DNA sample preparation kit (Illumina, USA) and sequenced with the Illumina HiSeq 2500 platform (MR DNA, USA) with 2 × 150-bp paired-end flow cells. A total of 6,373,398 read pairs (average read length, 150 bp; genome coverage, 389×) were quality filtered with FastQC and subsequently assembled *de novo* with SPAdes v. 3.9.0 with default parameters (6) to yield 37 contigs. The assembled contigs were scaffolded against the complete genome of E. xiangfangensis LMG 27195^T^ (GenBank accession number CP017183) with MeDuSa v. 1.6 with default parameters (7), and the final genome was annotated with the RAST server (8).

The complete genome sequence of E. xiangfangensis Pb204 comprises a circular chromosome and 2 small circular plasmids with a total size of 4,963,709 bp, a G+C content of 55.35%, and coding for 4,617 proteins. Phylogenomic classification of Pb204 was undertaken with digital DNA-DNA hybridization (dDDH) and average nucleotide identity (ANI) with the Genome-to-Genome Distance Calculator server v. 2.1 (9) and OrthoANI (10), respectively. The dDDH (76%) and ANI (97.12%) exceeded the proposed species boundary values (dDDH, >70%; ANI, >95 to 96%) (9, 10) for only 1 species, E. xiangfangensis, which suggests that Pb204 is a novel strain of this species.

**FIG 1:**
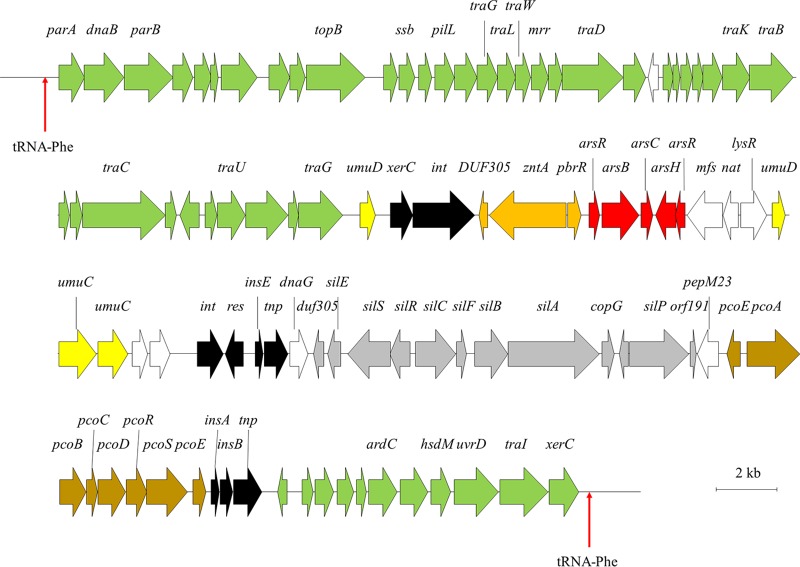
Diagrammatic representation of ICE*Exi*Pb204. Genes coding for proteins involved in silver resistance are indicated with gray arrows, copper resistance with brown arrows, arsenic resistance with red arrows, and zinc resistance with orange arrows. Genes involved in DNA damage SOS response (*umuDC*) are represented by yellow arrows and those coding for transposons and hypothetical proteins in black and white arrows, respectively. Proteins involved in ICE integration, excision, and maintenance are represented by green arrows.

The genome of E. xiangfangensis Pb204 is ∼300 kb larger than that of the type strain (E. xiangfangensis LMG 27195^T^; GenBank accession number CP017183), isolated from sourdough (11). This can largely be attributed to the presence of a ∼93.2-kb integrative and conjugative element (ICE) in Pb204 (ICE*Exi*Pb204). ICEs are highly diverse mobile genetic elements which integrate into and excise from bacterial replicons in a fashion analogous to that of both plasmids and bacteriophages (12). ICEs also carry cargo genes, which contribute to a wide range of phenotypes, including metabolic adaptation, antibiotic resistance, pathogenesis, and the production of secondary metabolites (12). ICE*Exi*Pb204 codes for 28 proteins that are involved in copper, silver, arsenic, and zinc resistance (Fig. 1). These resistance pathways have been suggested to be central to both gold resistance and the biomineralization of AuNPs (13, 14). It is therefore plausible that the ICE*Exi*Pb204-encoded heavy metal resistance pathways play a role in Au^3+^ uptake and subsequent reduction to AuNPs and may allow E. xiangfangensis Pb204 to produce other noble metal nanoparticles of biotechnological interest.

### Data availability.

The whole-genome sequence for E. xiangfangensis Pb204 has been deposited in DDBJ/EMBL/GenBank under the accession numbers CP030007 (chromosome), CP030008 (plasmid 1), and CP030009 (plasmid 2) (BioProject number PRJNA475446). The raw FASTQ reads have been deposited in the NCBI SRA database under the accession number SRX4936071.
